# Supplementary figures and images for: Experimental Crossing Confirms Reproductive Isolation between Cryptic Species within Eulimnogammarus verrucosus (Crustacea: Amphipoda) from Lake Baikal
Source: Int J Mol Sci. 2022 Sep 17;23(18):10858. doi: 10.3390/ijms231810858 (PMC9506054; doi:10.3390/ijms231810858)

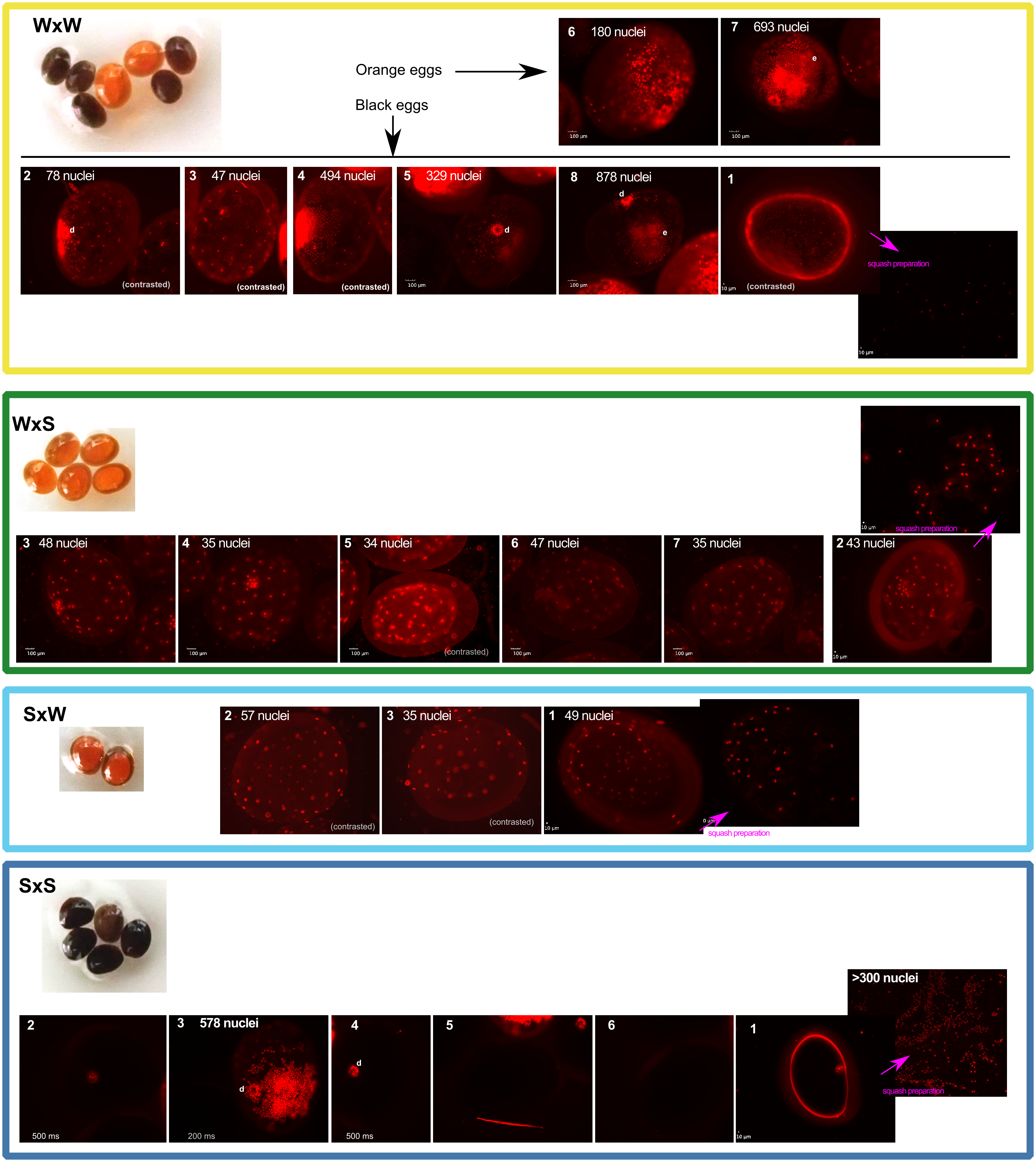

Supplement: Supplementary file 1 [file ijms-23-10858-s001.zip › ijms-1842672-supplementary/Figure S1 eggs.png]
